# Bridging Data Gaps in Healthcare: A Scoping Review of Transfer Learning in Structured Data Analysis

**DOI:** 10.34133/hds.0321

**Published:** 2025-09-03

**Authors:** Siqi Li, Xin Li, Kunyu Yu, Qiming Wu, Di Miao, Mingcheng Zhu, Mengying Yan, Yuhe Ke, Danny D’Agostino, Yilin Ning, Ziwen Wang, Yuqing Shang, Molei Liu, Chuan Hong, Nan Liu

**Affiliations:** ^1^ Centre for Quantitative Medicine, Duke-NUS Medical School, Singapore, Singapore.; ^2^ Department of Biostatistics and Bioinformatics, Duke University, Durham, NC, USA.; ^3^ Department of Anesthesiology, Singapore General Hospital, Singapore, Singapore.; ^4^ Department of Biostatistics, Columbia University Mailman School of Public Health, New York, NY, USA.; ^5^ Department of Biostatistics, Peking University Health Science Center, Peking University, Beijing, China.; ^6^ Beijing International Center for Mathematical Research, Peking University, Beijing, China.; ^7^ Programme in Health Services and Systems Research, Duke-NUS Medical School, Singapore, Singapore.; ^8^ Institute of Data Science, National University of Singapore, Singapore, Singapore.

## Abstract

**Background:** Clinical and biomedical research in low-resource settings often faces substantial challenges due to the need for high-quality data with sufficient sample sizes to construct effective models. These constraints hinder robust model training and prompt researchers to seek methods for leveraging existing knowledge from related studies to support new research efforts. Transfer learning (TL), a machine learning technique, emerges as a powerful solution by utilizing knowledge from pretrained models to enhance the performance of new models, offering promise across various healthcare domains. Despite its conceptual origins in the 1990s, the application of TL in medical research has remained limited, especially beyond image analysis. This review aims to analyze TL applications, highlight overlooked techniques, and suggest improvements for future healthcare research. **Methods:** Following the PRISMA-ScR guidelines, we conducted a search for published articles that employed TL with structured clinical or biomedical data by searching the SCOPUS, MEDLINE, Web of Science, Embase, and CINAHL databases. **Results:** We screened 5,080 papers, with 86 meeting the inclusion criteria. Among these, only 2% (2 of 86) utilized external studies, and 5% (4 of 86) addressed scenarios involving multi-site collaborations with privacy constraints. **Conclusions:** To achieve actionable TL with structured medical data while addressing regional disparities, inequality, and privacy constraints in healthcare research, we advocate for the careful identification of appropriate source data and models, the selection of suitable TL frameworks, and the validation of TL models with proper baselines.

## Introduction

Healthcare-related inequalities are particularly pronounced in low-resource settings, where conducting research is challenging due to poor quality and limited sample sizes of available data [1]. For example, in the Pan-Asian Resuscitation Outcomes Study (PAROS) [2] research network dataset, several key variables, such as public access defibrillation and post-resuscitation care hypothermia, are more commonly available in countries with established emergency medical services like Japan and South Korea, but less so in Thailand, Malaysia, or Turkey [2]. These disparities in data availability lead to challenges in robust modeling, as traditional solutions that rely on straightforward adoption of external models may not be applicable. There is a growing argument that researchers should prioritize recurring local validation of artificial intelligence (AI) models over external validation [3]. With the rapid emergence of AI-based solutions, researchers are exploring ways to enhance the quality of local model development and validation by borrowing and adapting external information to support research and applications in low-resource settings. Efficiently incorporating and transferring existing information into new studies is crucial for adequately addressing local needs and conditions.

Transfer learning (TL) is a learning paradigm that leverages knowledge from a related source domain (Box 1) to enhance learning or modeling performance in a target domain (Box 1) [4]. For instance, transferring the knowledge of a resuscitation outcome prediction model involves using the PAROS dataset from country A as the source domain and country B as the target domain. In this example, the study designs (outcome, data collection process, target populations, etc.) of the source and target domains are comparable (Box 1) since they both focus on the same outcome of return of spontaneous circulation and target populations who can experience out-of-hospital cardiac arrest. In other situations, the study designs of source and target domains can differ significantly, such as data collected for different diseases [5,6], treatments [7], biological samples [8,9], and patient groups [10,11]. For example, in cancer drug sensitivity prediction, the source domain could be data from one group of patients diagnosed with cancer C, and the target domain could be data from another group of patients diagnosed with cancer D. TL is particularly beneficial in low-resource settings, where using external information can help overcome challenges posed by factors such as physician shortages and limited infrastructure resources [12]. However, the technology is also beneficial in other research scenarios, improving the quality and efficiency of studies in various healthcare settings by utilizing external knowledge from related studies.

Box 1 Transfer learning (TL) glossary

**Source data/domain:** the original dataset used to train a model before it is applied to new data.

**Target data/domain:** the new dataset of interest used to train new models.

**Source model:** the original model trained using the source data/domain.

**Target model:** the new model trained using only the target data/domain.

**TL model:** the model trained using TL techniques.

**Domain similarity:** the degree of similarity between the study designs of source and target data. Examples include:

o Comparable: source and target domains are both patient vital signs collected at emergency departments.
o Different diseases: source and target domains are both patient medical records, but one pertains to patients diagnosed with periodontal disease, while the other pertains to patients diagnosed with rheumatoid arthritis.
o Different diseases and treatments: source and target domains are both drug sensitivity data, but one is for cisplatin in multiple myeloma patients, and the other is for docetaxel in breast cancer patients.
o Different types of samples: source and target domains are both gene expression data but collected from different tissues.
o Different settings: source and target domains are both intensive care unit (ICU) data but measured with different ICU monitoring systems.
o Different patients: source and target domains are both EHR surgical data but involve patients with different surgical complications.

**TL types:**

o Parameter transfer: leverage parameters from the source model to update and fine-tune the target model using target data.
o Feature representation: embed the good feature representations from source model to the target.
o Instance reweighting: compare the similarity between instances from source and target data and minimize model loss through weighted instances.

**Source-free TL:** the process of TL where only the source model is used, without the source data.

**Temporal adaptation:** the process of TL where knowledge transfer is conducted over time.

The concept of TL was first introduced in 1995 [13] to address the need for machine learning (ML) algorithms that can retain and reuse previously learned knowledge [14]. Despite its early emergence, TL has yet to fully realize its potential in analyzing structured healthcare data [15], such as electronic health records (EHRs) and data from traditional cohort studies [16]. Existing reviews on TL applications, whether in general domains or healthcare, primarily focus on methods developed by computer scientists, neglecting those from statistical domains [14,15,17–19]. This oversight has led to the underutilization of many statistical TL approaches, resulting in limitations for studies requiring more rigorous statistical analysis. A thorough examination of a broader range of TL methods could provide valuable insights for their future integration and application in the field. Additionally, the current literature [15,19] lacks comprehensive discussions on the diverse scenarios of TL applications in healthcare, particularly in cross-institutional collaborations and scenarios complicated by data privacy constraints.

The aim of this review is to (a) analyze how TL can be applied to various types of clinical and biomedical research using structured data, (b) spotlight important TL techniques, including those that have been previously overlooked or under-analyzed in the healthcare domain, and (c) provide suggestions for improving future research practices.

## Materials and Methods

### Search strategy and selection criteria

This scoping review was conducted based on the PRISMA-ScR guidelines [20]. It was not preregistered on platforms such as the Open Science Framework. We conducted a search for published articles that employed TL with structured clinical or biomedical data. We searched SCOPUS, MEDLINE, Web of Science, Embase, and CINAHL databases for articles published by 2025 January 3, utilizing a combination of search terms, including “electronic health records”, “EHR”, “electronic medical records”, “EMR”, “registry/registries”, “tabular”, “gene”, “bioassay”, “biological network”, “transfer learning”, “domain adaptation”, and “knowledge transfer”. Only articles published in English were included. A detailed search strategy is presented in Table S1.

The final search was conducted on 2025 January 3. After removing duplicates, each article was independently screened by 2 reviewers (selected from S.L., X.L., K.Y., Q.W., and D.M.) based on titles and abstracts, with a third reviewer resolving conflicts. Publications selected in the initial screening underwent full-text examination to ensure they met the following inclusion criteria: (a) using structured clinical or biomedical data to address research questions directly relevant to clinical or biomedical research, (b) employing TL with identifiable source and target data as well as source and target models [21,22], (c) being an original research article (excluding review, lecture notes, and protocol papers for R or Python packages), and (d) being peer-reviewed (excluding conference proceedings, preprints, reports, and dissertations) with full-text availability. The extracted data were synthesized qualitatively to identify key trends, methodologies, and gaps.

### Data extraction

Full-text information extraction was conducted by each reviewer and double-checked by at least one other reviewer. We extracted information from the selected publications from 3 perspectives: study characteristics (study field, origin of reused model, participating regions, data public availability, data types, outcomes, number of participating sources and targets, number of features, sample size, study type, validation of TL model, and code availability), modeling characteristics (types of task, modeling approach, hyperparameter methods, and model performance metrics), and TL characteristics (capability of privacy-preserving, types of TL, and solution for covariate effect heterogeneity).

## Results

**Fig. 1.**
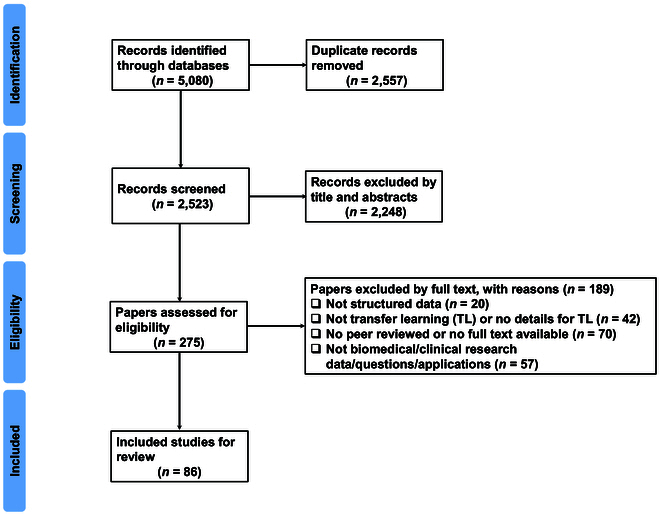
Preferred reporting items for scoping reviews (PRISMA-ScR) flow diagram.

**Fig. 2.**
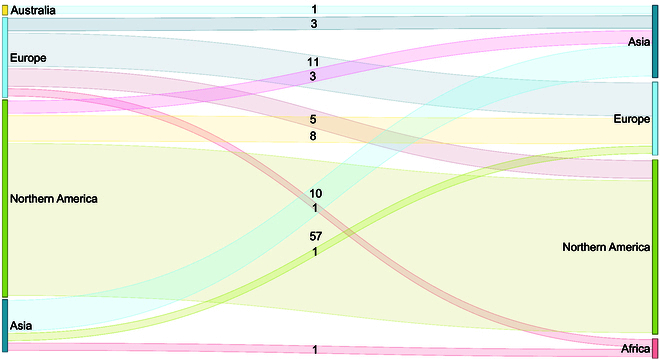
Sankey diagram illustrating the counts of matches between source and target regions.

Our search yielded 5,080 articles. After removing 2,557 duplicate records, 2,523 articles were screened based on their titles and abstracts. Of these, 275 articles were selected for full-text screening, and 86 articles were ultimately included in our review. Figure 1 presents the PRISMA flow diagram, detailing the selection process. Notably, 78 of the 86 reviewed papers were published in 2020 or later, indicating a recent surge of interest in TL for clinical research with structured data. As shown in Table 1, cancer medicine is the most investigated field for TL techniques, with a total of 23 papers. Additionally, in approximately 27% (23 of 86) of the papers, the outcome types for both the source and target models are binary. In Fig. 2, we present a Sankey diagram that maps the origins of both source and target data in each study. The diagram highlights that most knowledge transfer occurs between source and target data originating from developed regions, rather than involving regions with fewer research resources.

**Table 1.** Summary of information extraction table

Model characteristics	No. of papers	Examples
Task		
Prediction	79	[27,36,45]
Nonprediction	2	[46,47]
Both prediction and nonprediction	5	[10,23,48]
Model type		
Statistical regression
(1) Logistic regression	5	[49–51]
(2) Lasso	3	[37,38,52]
(3) Latent class regression	1	[53]
(4) Elastic net	2	[54,55]
(5) Cox regression	2	[23,41]
(6) Bayesian negative binomial model	1	[56]
(7) Generalized linear model	5	[57–59]
(8) Hierarchical Bayesian linear model	1	[60]
(9) Hierarchical infinite Bayesian latent factor regression	1	[10]
Neural networks		
(1) Multilayer perceptron	8	[9,27,61]
(2) Recurrent neural network	7	[25,62,63]
(3) Autoencoder	4	[24,64,65]
(4) Convolutional neural network	2	[5,66]
(5) DeepSurv	1	[42]
(6) Generative adversarial network	1	[8]
(7) Graph neural network	2	[11,67]
(8) Transformers	3	[68–70]
(9) Other	12	[71–73]
Gradient boosting machine	2	[36,74]
Random forest	2	[75,76]
Gaussian graphical	2	[46,77]
Gaussian process	1	[78]
Matrix factorization	1	[47]
Support vector machine	1	[79]
Multiple	15	[80–82]
Not available	1	[83]
TL characteristics		
Types of TL		
Parameter transfer	61	[5,23,45]
Instance reweighting	10	[24,36,51]
Feature representation	8	[7,8,84]
Multiple	7	[74,80,85]
Capable of preserving privacy		
Yes	51	[23,86,87]
No	35	[24,36,74]
Strategy for heterogeneity in covariate effects		
Yes	15	[38,48,77]
No	71	[27,36,45]
Study characteristics		
Overall study field		
Administrative/education	3	[54,68,81]
Cancer medicine	23	[5,23,79]
Cardiovascular health	1	[27]
Emergency medicine	11	[24,26,45]
Drugs	8	[7,8,76]
Endocrine	5	[28,88,89]
Gastroenterology	1	[67]
Genomics	8	[52,90,91]
Infectious disease	3	[41,61]
Internal medicine	2	[62,92]
Neurology	3	[60,83,93]
Ophthalmology	1	[57]
Pediatric	2	[47,94]
Perioperative	1	[10]
Precision medicine	10	[71,95,96]
Renal	4	[36,49,74]
Outcome type (source; target)		
(Binary; binary)	42	[25,49,50]
(Survival; survival)	7	[23,24,45]
(Continuous; continuous)	10	[27,60,80]
(Multi-category; multi-category)	3	[71,75,82]
(Time series; time series)	1	[42]
Different pairs	9	[79,86,97]
Semi-supervised	6	[58,98,99]
Not available	8	[26,54,77]
Origin of reused model		
Current study	84	[36,45,86]
External source	2	[27,28]
Comparison with (no-TL) models		
Yes	71	[27,45,74]
No	15	[36,82,86]
Data public availability		
Yes	52	[42,48,60]
No	34	[25,26,54]
Code availability		
Yes	42	[23,27,86]
No	44	[24,36,45]

### Data and study characteristics

**Fig. 3.**
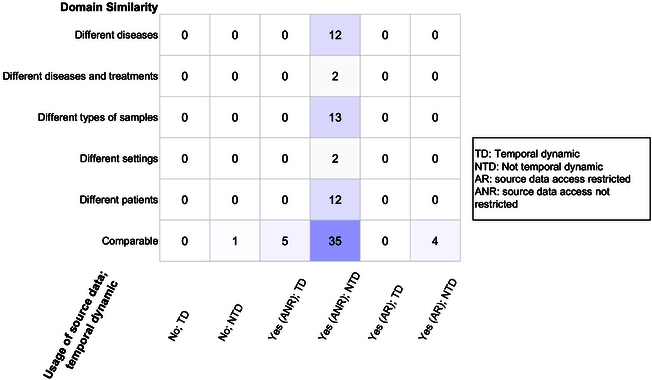
Comprehensive characteristic analysis for included TL articles categorized from clinical perspectives.

We classify the articles based on their clinical types of TL studies from 3 perspectives, as shown in Fig. 3. First, domain similarity (Box 1) includes 6 different scenarios observed in the reviewed studies: comparable [23,24], different diseases [5,6], different diseases and treatments [7], different types of samples [8,9], different patient groups [10,11], and different settings [25,26]. Second, the usage of source data considers whether the TL process involves only the usage of the source model without using source data or if source data are used. When source data are used, we examine whether the access to source data is restricted due to privacy constrains, such as when the source data are kept by owners who cannot share it with third parties. Third, temporal adaptation (Box 1) considers whether the TL between source and target data involves models that evolve over time.

Both single and multiple source domains are represented in the studies included in our review, with single source domains being the majority (78%, 67 of 86). Notably, 88% (76 of 86) of the included papers conduct TL using source data with unrestricted access, while only 2 studies [27,28] utilize source models obtained from external studies without using any source data. Additionally, 52% (45 of 86) of TL instances in the included papers occur between source and target domains that are comparable. Only 6% (5 of 86) of the reviewed articles involve TL with temporal adaptations.

### Modeling

In this study, we categorize modeling into 2 types: prediction and nonprediction, based on whether the primary goal is predicting outcomes or nonprediction tasks such as investigating the relationship between covariates and outcomes or conducting phenotyping tasks [16]. For example, the study by Edmondson et al. [29] is considered as an association study, since it focused on investigating the association between length of stay in COVID-19 patients with patient characteristics. As shown in Table 1, all articles included in the review involve prediction tasks, with only 4 of them additionally conducting nonprediction tasks. We also summarized the types of models used by these articles, with 40 of the 86 papers involving neural networks (NNs) and 21 utilizing traditional statistical models. A total of 15 studies conducted modeling using more than one type of model.

### TL techniques

**Fig. 4.**
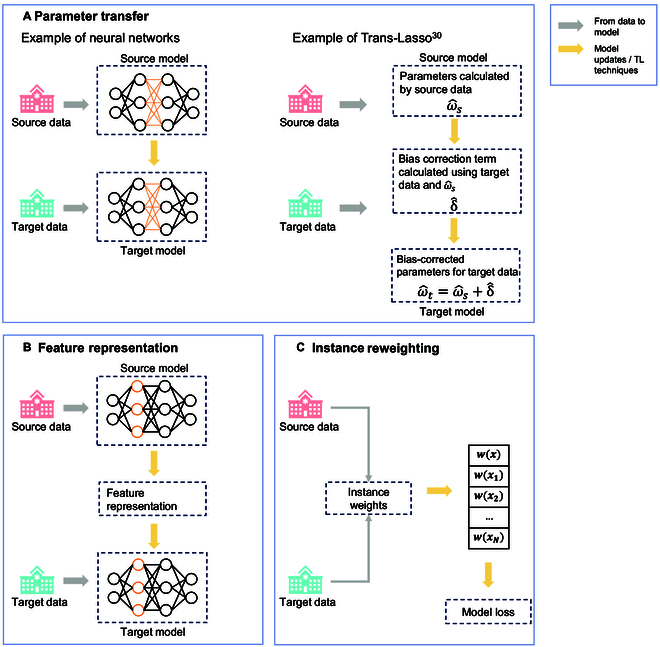
Illustration of 3 types of TL methods. (A) Parameter transfer: Model parameters learned from the source data are adapted using target data to improve performance. (B) Feature representation: Transferable knowledge is encoded in shared feature representations to enable generalization across domains. (C) Instance reweighting: Source instances are reweighted based on their similarity to target instances to reduce distribution mismatch.

We classified TL methods into 3 categories based on specific knowledge transfer techniques: parameter transfer, feature representation, and instance reweighting. As detailed in Fig. 4, parameter transfer (Box 1) involves using parameters from the source model to update and fine-tune the target model using target data. Feature representation (Box 1) embeds the feature representation from the source model to the new model. Instance reweighting (Box 1) compares the similarity between instances from source and target data, minimizing model loss through weighted instances. Among the 86 studies reviewed, 61 (71%) employed parameter transfer, 8 (9%) used feature representation, 10 (12%) focused on instance reweighting, and 7 (8%) applied more than one type of TL strategy.

We also summarize TL methods in this review regarding their ability to preserve privacy. Source-free [30] TL (Box 1) uses only source models without any source data, making it privacy-preserving. Most parameter transfer TL methods can preserve privacy, while most instance reweighting TL methods cannot. Detailed information on the included papers is provided in the full information extraction tables in Tables S2 to S4. As summarized in Table 1, 51 of 86 included papers utilized privacy-preserving TL methods. Additionally, we evaluate the capability of TL methods to handle heterogeneity in covariate effects (see more details in Discussion), with 15 of the 86 included papers employing such methods.

In Table 2, we provide an overview of the TL frameworks reviewed in this paper, focusing on their applicability across 3 different scenarios related to access to and usage of source data and model types: (a) using source data without access restrictions, such as publicly available data; (b) using source data with access restrictions, such as data owned by collaborators with privacy constraints that cannot be shared; and (c) not using source data but instead utilizing only the source model. Table 2 can serve as a reference for users interested in applying TL frameworks in the future, providing recommendations on which framework to choose based on the different data scenarios and types of models. It is important to note that the summarized usage presented here is specific to this review, and since some TL methods are model-agnostic, their applicability may extend beyond the modeling scenarios outlined in the table.

**Table 2.** Summary of TL frameworks reviewed in this paper, categorized by their applicability across different data scenarios and model types

TL framework	Use source data (without access restriction)	Use source data (with access restriction)	Without source data
Statistical regression			
Logistic regression with or without penalty	COMMUTE [38] ^a^, DRAMATIC [28] ^a^, TR-CEN [54], TL-Multi [52] ^a^, Trans-Lasso [37] ^a^, TRANSACT [55], PMTL [49]	COMMUTE [38] ^a^, DRAMATIC [28] ^a^, TL-Multi [52] ^a^, Trans-Lasso [37] ^a^, TRANSACT [55]	COMMUTE [38] ^a^, DRAMATIC [28] ^a^, TL-Multi [52] ^a^, Trans-Lasso [37] ^a^, TRANSACT [55]
Cox regression	SurvMaximin [41] ^a^, Trans-Cox [23] ^a^	SurvMaximin [41] ^a^, Trans-Cox [23] ^a^	SurvMaximin [41] ^a^, Trans-Cox [23] ^a^
Generalized linear model	FETA [59] ^a^, STEAM [58], STRIFLE [98] ^a^, TransHDGLM [48] ^a^	FETA [59] ^a^, STEAM [58], STRIFLE [98] ^a^, TransHDGLM [48] ^a^	FETA [59] ^a^, STEAM [58], STRIFLE [98] ^a^, TransHDGLM [48] ^a^
Bayesian methods	ebTL [60], HIFM [10] ^a^, OBTL [56]	ebTL [60], HIFM [10] ^a^, OBTL [56]	HIFM [10] ^a^, OBTL [56]
Neural network			
Multilayer perceptron	CSG2A [55], deep LDL-HER [27], DREDDA [74], SCAD [9]	CSG2A [55], deep LDL-HER [27]	CSG2A [55], deep LDL-HER [27]
Graph neural network	DQSurv [7], Gdmicro [67]	DQSurv [7]	DQSurv [7]
Autoencoder	Drug2TLE [65], HRU control [24], VAECox [64]	VAECox [64]	VAECox [64]
Transformer	Claim-PT [68]	Claim-PT [68]	Claim-PT [68]
Generative adversarial network	AITL [8]		
Other	ERR-DGN [88], HDTL-SRP [93], microDELTA [92], PDSP [100], SBINN [87], scCaT [101], TCAP [97], Velodrome [73]	ERR-DGN [88], HDTL-SRP [93], microDELTA [92], SBINN [87], scCaT [101], TCAP [97], Velodrome [73]	ERR-DGN [88], HDTL-SRP [93], microDELTA [92], SBINN [87], scCaT [101], TCAP [97], Velodrome [73]
Others			
Gradient boosting machine	HA-Boost [36], TransferGBM [74]	TransferGBM [74]	TransferGBM [74]
Gaussian graphical model	Trans-CLIME [77] ^a^	Trans-CLIME [77] ^a^	Trans-CLIME [77] ^a^
Random forest	DeepMicroCancer [75]		

^a^ TL methods that are claimed to be able to handle model heterogeneity.

## Discussion

While TL has been widely researched in the general AI field, leading to successful applications in text classification, image processing, and audio analysis across various domains such as transportation and recommender systems [17,31,32], its application in healthcare research using structured data remains underexplored. Our review highlights several key challenges and opportunities for TL in healthcare, which are important for advancing research and practice in this field.

Our review found that existing research has not fully tapped into TL’s potential to assist in low-resource settings. This is evident as many applications require access to source data, which poses a significant challenge for low-resource environments where directly utilizing models from published works without source data is often the only viable option. Additionally, we noticed that many studies utilize TL where source data are freely accessible, while applications in cross-site collaborations with privacy constraints are limited. Furthermore, many studies lack sufficient evidence demonstrating the generalizability of their TL methods to different scenarios, especially concerning the degree of domain similarity. In this section, we provide a detailed discussion on how to fully leverage TL in low-resource settings, explore additional benefits of TL for privacy-preserving multi-site collaborations, and address technical details specific to structured clinical and biomedical data.

In the following subsections, we discuss how to fully leverage TL in low-resource settings, explore its benefits for privacy-preserving multi-site collaborations, and examine the technical aspects specific to structured clinical and biomedical data.

### Leveraging TL in low-resource settings and beyond

TL can occur in various scenarios. As shown in Fig. 3, most existing TL applications have been conducted between source and target domains with comparable study designs. However, several gaps remain in other scenarios, indicating untapped potential beyond the scope of the studies reviewed. For instance, there are only 5 papers that explore temporal dynamics, and all of them involve comparable source and target data, with no consideration of other scenarios—indicating a significant gap in research. Additionally, while TL with temporal adaptation shows promise, its application is still limited and warrants further investigation.

One key advantage of TL is its ability to leverage existing knowledge from published works, as demonstrated by Hwang et al.’s study [27] and Zhou et al.’s study [28], the only 2 examples among the 86 papers reviewed. This application allows data owners to create new models using publicly available resources, which is especially promising in scenarios where fostering collaborations is challenging and time-consuming for less established researchers. Furthermore, the TL methods included in this review can generally be implemented with standard computing environments, making them accessible even in low-resource settings with limited computational power.

As shown in Fig. 2, most knowledge transfer occurred from developed regions, highlighting the existing disparities in clinical studies using ML methods across the globe [33]. These disparities are often driven by financial factors, which contribute to the limited availability of high-quality data for modeling in resource-limited settings [1]. By using source models from published works, researchers can circumvent issues associated with data sharing and collaboration when public source data are unavailable. Consequently, TL has the potential to bridge regional disparities in resource-limited settings.

### Privacy-preserving with source-free TL

Building on the advantages of TL for low-resource settings, its role in privacy-preserving scenarios of research collaborations is another critical aspect. With the rise of cross-site collaborations in healthcare research, privacy preservation has become increasingly significant due to data-sharing constraints. Federated learning (FL) has emerged as a key technique for collaborative model training without data sharing [34,35]. Unlike FL, source-free TL can utilize existing information—such as model parameters from published works—to update individual models without a co-training process. This approach streamlines model training and also allows the incorporation of external information into new studies without reanalyzing source data. Therefore, source-free TL frameworks can achieve functions similar to FL and can be integrated with FL to further leverage federated models for client-specific model personalization and calibration.

### Heterogeneity between source and target domains

Having discussed the potential of TL for low-resource setting and multi-site collaboration, we now delve into the capability of TL to handle varying degrees of domain similarity. Similar to the scenarios of FL [16], traditional TL techniques address data heterogeneity, broadly defined as scenarios where data are not independently and identically distributed [24,36]. In statistical literature, heterogeneity between source and target domains is usually categorized into heterogeneity in covariate effects (the conditional distribution of Y∣X) and heterogeneity in covariate distributions ($P\left(X\right)$). While most TL methods can handle heterogenous covariate distributions, their performance in the presence of covariate effect heterogeneity has not been thoroughly examined.

TL methods such as Trans-Lasso [37], Trans-Cox [23], and COMMUTE [38] typically conceptualize a calibration term to address covariate effect heterogeneity. In this review, 15 of 86 papers have employed such methods, demonstrating their capability to handle covariate effect heterogeneity primarily through simulation studies. In addition, although not utilized in the 86 papers included in this review, Arjovsky et al. [39] proposed invariant risk minimization (IRM), which adds a penalty term to enforce invariant feature representations across different environments. This encourages model robustness and improves generalization, particularly in out-of-distribution scenarios.

Understanding how TL methods perform under different levels of domain similarity is crucial. For TL methods evaluated using limited datasets, confidence in their generalizability to different TL and research settings is lower. Researchers may consider benchmarking various TL frameworks across different simulation scenarios and real-world data examples to provide a clearer understanding of their robustness and applicability.

### Suggested steps for applications

**Fig. 5.**
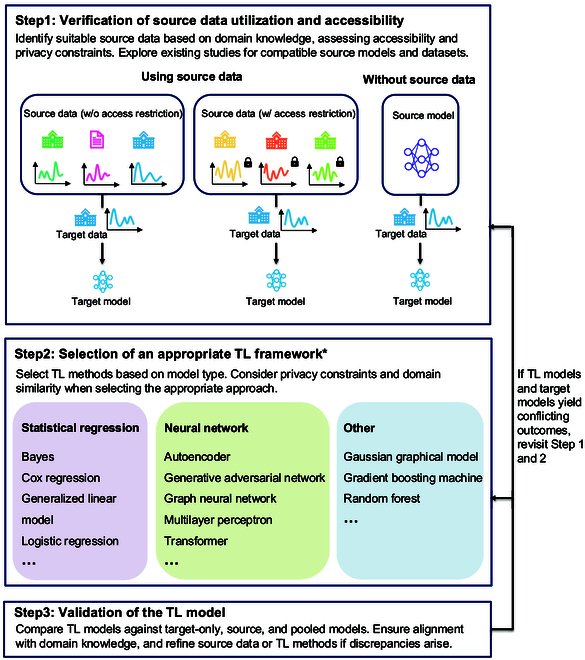
Suggestions for future users.

In this section, we introduce our own suggestions for future research intending to apply TL for structured clinical and biomedical data. A summary of the ensuing discussion is outlined in Fig. 5, which further visualizes these processes in detail.

The first step for user is to identify available and appropriate source domains. If source data will not be used, the source typically comes from existing studies, such as Hwang et al. [27] adapting a deep neural network (DNN) from Lee et al. [40]. If the source data are fully available, the TL process can proceed straightforwardly, as seen in Zhang et al. [36] using affiliated hospital data, Li et al. [37] employing public datasets, and Gu et al. [38] utilizing collaborator-shared data. When access to source data is restricted, privacy-preserving TL methods should be applied, as demonstrated by Wang et al. [41] and Zhu et al. [42]. Successful TL preparation involves identifying relevant studies and assessing potential source data, including public datasets or collaborator-owned data, while considering privacy constraints.

The second step is to use TL framework that suits the research question. The choice of TL frameworks depends on modeling needs and privacy constraints. Parameter transfer methods, commonly used for NNs and traditional statistical regressions, are generally privacy-preserving, while many instance reweighing TL methods, such as those by Zhang et al. [36] and Zhu and Pu [24], are not, since they involve calculating the distance between source and target data. TL often overlaps with FL in multi-site collaborations, where privacy-preserving methods are essential [41]. Additionally, TL frameworks should be chosen based on their ability to handle heterogeneity in covariate effects, ensuring adaptability to differences in study designs between source and target domains.

The third step is to validate TL models with proper baselines. Effective evaluation of TL models relies heavily on adequate baseline comparisons. The choice of evaluation metrics should align with the specific model and task, ensuring consistency with both the source and target models, with common metrics including area under the receiver operating characteristic curve (AUROC) for binary classification, mean squared error (MSE) for continuous variable predictions, and confidence intervals for odds ratios, among others. As shown in Table 1, most studies (71 of 86) included non-TL models, with target and source models commonly serving as baselines (Fig. 5). It is crucial to ensure that TL models do not yield conflicting outcomes, especially when compared to target-only models. A performance drop in prediction tasks or reduced confidence in association studies may signal issues with TL methods or the relevance of the source data. If discrepancies arise, users should revisit earlier steps, consult experts, and refine the source data selection or TL techniques. When source data are accessible, pooled models can serve as an additional baseline for validation.

### Limitations

This review provides a comprehensive overview of TL in tabular biomedical and clinical data but does not systematically include technical conference papers or other gray literature, as these are not fully indexed in standard medical databases. While this approach ensures methodological consistency by adhering to the PRISMA-ScR guidelines for scoping reviews, it may exclude some cutting-edge advancements in TL that are primarily disseminated through conferences. Researchers interested in more technical TL methodologies may explore conference-specific search engines like OpenReview [43] for additional insights [44] beyond this review.

## Conclusion

This review offers a comprehensive summary of TL techniques and their applications for structured medical data, categorizing them from clinical and medical perspectives. TL holds significant promise for enhancing model performance in low-resource settings by leveraging preexisting knowledge. However, most studies only use data from developed regions, highlighting a gap in addressing resource inequalities. Additionally, privacy-preserving TL methods are underutilized. By carefully selecting source domains, employing suitable TL frameworks, and validating models against proper baselines, TL can improve healthcare outcomes and address disparities. Future research should focus on developing robust, privacy-preserving TL methods to expand their applicability in diverse healthcare contexts.

## References

[1] Van Zyl C, Badenhorst M, Hanekom S, Heine M. Unravelling ‘low-resource settings’: A systematic scoping review with qualitative content analysis. BMJ Glob Health. 2021;6(6):e005190. 10.1136/bmjgh-2021-005190 PMC8183220 34083239

[2] Ong MEH, Shin SD, Tanaka H, Ma MHM, Khruekarnchana P, Hisamuddin N, Atilla R, Middleton P, Kajino K, Leong BSH, et al. Pan-Asian resuscitation outcomes study (PAROS): Rationale, methodology, and implementation. Acad Emerg Med. 2011;18(8):890–897. 21843225 10.1111/j.1553-2712.2011.01132.x

[3] Youssef A, Pencina M, Thakur A, Zhu T, Clifton D, Shah NH. External validation of AI models in health should be replaced with recurring local validation. Nat Med. 2023;29(11):2686–2687. 37853136 10.1038/s41591-023-02540-z

[4] Zhuang F, Qi Z, Duan K, Xi D, Zhu Y, Zhu H, Xiong H, He Q. A comprehensive survey on transfer learning. Proc IEEE. 2020;109(1):43–76.

[5] Alrefai N, Ibrahim O, Shehzad HMF, Altigani A, Abu-ulbeh W, Alzaqebah M, Alsmadi MK. An integrated framework based deep learning for cancer classification using microarray datasets. J Ambient Intell Humaniz Comput. 2023;14(3):2249–2260.

[6] Hanczar B, Bourgeais V, Zehraoui F. Assessment of deep learning and transfer learning for cancer prediction based on gene expression data. BMC Bioinformatics. 2022;23(1):262. 35786378 10.1186/s12859-022-04807-7 PMC9250744

[7] Turki T, Wang JT. Clinical intelligence: New machine learning techniques for predicting clinical drug response. Comput Biol Med. 2019;107:302–322. 30771879 10.1016/j.compbiomed.2018.12.017

[8] Sharifi-Noghabi H, Peng S, Zolotareva O, Collins CC, Ester M. AITL: Adversarial inductive transfer learning with input and output space adaptation for pharmacogenomics. Bioinformatics. 2020;36(Suppl-1):i380–i388. 32657371 10.1093/bioinformatics/btaa442 PMC7355265

[9] Zheng Z, Chen J, Chen X, Huang L, Xie W, Lin Q, Li X, Wong K-C. Enabling single-cell drug response annotations from bulk RNA-seq using SCAD. Adv Sci. 2023;10(11):2204113. 10.1002/advs.202204113 PMC10104628 36762572

[10] Lorenzi E, Henao R, Heller K. Hierarchical infinite factor models for improving the prediction of surgical complications for geriatric patients. Ann Appl Stat. 2019;13(4):2637–2661.

[11] Duan M, Wang Y, Zhao D, Liu H, Zhang G, Li K, Zhang H, Huang L, Zhang R, Zhou F. Orchestrating information across tissues via a novel multitask GAT framework to improve quantitative gene regulation relation modeling for survival analysis. Brief Bioinform. 2023;24(4):bbad238. 37427963 10.1093/bib/bbad238

[12] Petroze RT, Byiringiro JC, Ntakiyiruta G, Briggs SM, Deckelbaum DL, Razek T, Riviello R, Kyamanywa P, Reid J, Sawyer RG, et al. Can focused trauma education initiatives reduce mortality or improve resource utilization in a low-resource setting? World J Surg. 2015;39(4):926–933. 25479817 10.1007/s00268-014-2899-y PMC4700401

[13] Wu X, Kumar V, Ross Quinlan J, Ghosh J, Yang Q, Motoda H, McLachlan GJ, Ng A, Liu B, Yu PS, et al. Top 10 algorithms in data mining. Knowl Inf Syst. 2008;14(1):1–37.

[14] Pan SJ, Yang Q. A survey on transfer learning. IEEE Trans Knowl Data Eng. 2009;22(10):1345–1359.

[15] Ebbehoj A, Thunbo MØ, Andersen OE, Glindtvad MV, Hulman A. Transfer learning for non-image data in clinical research: A scoping review. PLOS Digital Health. 2022;1(2): Article e0000014. 36812540 10.1371/journal.pdig.0000014 PMC9931256

[16] Li S, Liu P, Nascimento GG, Wang X, Leite FRM, Chakraborty B, Hong C, Ning Y, Xie F, Teo ZL, et al. Federated and distributed learning applications for electronic health records and structured medical data: A scoping review. J Am Med Inform Assoc. 2023;30(12):2041–2049. 37639629 10.1093/jamia/ocad170 PMC10654866

[17] Zhuang F, Qi Z, Duan K, Xi D, Zhu Y, Zhu H, Xiong H, He Q. A comprehensive survey on transfer learning. Proc IEEE. 2021;109:43–76.

[18] Kaboli M. A review of transfer learning algorithms. Technische Universität München; 2017.

[19] Wan Z, Yang R, Huang M, Zeng N, Liu X. A review on transfer learning in EEG signal analysis. Neurocomputing. 2021;421:1–14.

[20] Tricco AC, Lillie E, Zarin W, O’Brien KK, Colquhoun H, Levac D, Moher D, Peters MDJ, Horsley T, Weeks L, et al. PRISMA extension for scoping reviews (PRISMA-ScR): Checklist and explanation. Ann Intern Med. 2018;169(7):467–473. 30178033 10.7326/M18-0850

[21] Zhuang F, Qi Z, Duan K, Xi D, Zhu Y, Zhu H, Xiong H, He Q. A comprehensive survey on transfer learning. Proc IEEE. 2021;109(1):43–76.

[22] Ebbehoj A, Thunbo MØ, Andersen OE, Glindtvad MV, Hulman A. Transfer learning for non-image data in clinical research: A scoping review. PLOS Digital Health. 2022;1(2): Article e0000014. 36812540 10.1371/journal.pdig.0000014 PMC9931256

[23] Li Z, Shen Y, Ning J. Accommodating time-varying heterogeneity in risk estimation under the Cox model: A transfer learning approach. J Am Stat Assoc. 2023;118(544):2276–2287. 38505403 10.1080/01621459.2023.2210336 PMC10950074

[24] Zhu S, Pu J. A self-supervised method for treatment recommendation in sepsis. Front Inform Technol Electron Eng. 2021;22(7):926–939.

[25] Shickel B, Davoudi A, Ozrazgat-Baslanti T, Ruppert M, Bihorac A, Rashidi P. Deep multi-modal transfer learning for augmented patient acuity assessment in the intelligent ICU. Front Digit Health. 2021;3:640685. 33718920 10.3389/fdgth.2021.640685 PMC7954405

[26] Yang B, Ye M, Tan Q, Yuen PC. Cross-domain missingness-aware time-series adaptation with similarity distillation in medical applications. IEEE Trans Cybern. 2020;52(5):3394–3407. 10.1109/TCYB.2020.3011934 32795976

[27] Hwang S, Gwon C, Seo DM, Cho J, Kim JY, Uh Y. A deep neural network for estimating low-density lipoprotein cholesterol from electronic health records: Real-time routine clinical application. JMIR Med Inform. 2021;9(8): Article e29331. 34342586 10.2196/29331 PMC8371492

[28] Zhou D, Liu M, Li M, Cai T. Doubly robust augmented model accuracy transfer inference with high dimensional features. J Am Stat Assoc. 2025;120(549):524–534. 40302926 10.1080/01621459.2024.2356291 PMC12037143

[29] Edmondson MJ, Luo C, Nazmul Islam M, Sheils NE, Buresh J, Chen Z, Bian J, Chen Y. Distributed quasi-Poisson regression algorithm for modeling multi-site count outcomes in distributed data networks. J Biomed Inform. 2022;131:104097. 35643272 10.1016/j.jbi.2022.104097 PMC11874216

[30] Kundu JN, Venkat N, Babu RV*.* Universal source-free domain adaptation. Paper presented at: 2020 IEEE/CVF Conference on Computer Vision and Pattern Recognition, CVPR 2020; 2020 Jun 13–19; Seattle, WA, USA.

[31] Iman M, Arabnia HR, Rasheed K. A review of deep transfer learning and recent advancements. Technologies. 2023;11(2):40.

[32] Niu S, Liu Y, Wang J, Song H. A decade survey of transfer learning (2010–2020). IEEE Trans Artif Intell. 2020;1(2):151–166.

[33] Yang R, Nair SV, Ke Y, D’Agostino D, Liu M, Ning Y, Liu N. Disparities in clinical studies of AI enabled applications from a global perspective. NPJ Digit Med. 2024;7(1):209. 39127820 10.1038/s41746-024-01212-7 PMC11316833

[34] Choudhury O, Gkoulalas-Divanis A, Salonidis T, Sylla I, Park Y, Hsu G, Das A. Differential privacy-enabled federated learning for sensitive health data. arXiv. 2019. 10.48550/arXiv.1910.02578 PMC7153050 32308824

[35] Islam H, Alaboud K, Paul T, Rana MKZ, Mosa A. A privacy-preserved transfer learning concept to predict diabetic kidney disease at out-of-network siloed sites using an in-network federated model on real-world data. AMIA Summits Transl Sci Proc. 2022;2022:264. 35854714 PMC9285167

[36] Zhang X, Liu K, Yuan B, Wang H, Chen S, Xue Y, Chen W, Liu M, Hu Y. A hybrid adaptive approach for instance transfer learning with dynamic and imbalanced data. Int J Intell Sys. 2022;37(12):11582–11599. 10.1002/int.23055 PMC9936919 36816520

[37] Li S, Cai TT, Li H. Transfer learning for high-dimensional linear regression: Prediction, estimation and minimax optimality. J R Stat Soc Series B Stat Methodol. 2022;84(1):149–173. 35210933 10.1111/rssb.12479 PMC8863181

[38] Gu T, Lee PH, Duan R. COMMUTE: Communication-efficient transfer learning for multi-site risk prediction. J Biomed Inform. 2023;137: Article 104243. 36403757 10.1016/j.jbi.2022.104243 PMC9868117

[39] Arjovsky M, Bottou L, Gulrajani I, Lopez-Paz D*.* Invariant risk minimization. arXiv. 2020. 10.48550/arXiv.1907.02893

[40] Lee T, Kim J, Uh Y, Lee H. Korean public and hospital data for estimating LDL-cholesterol. Data Brief. 2019;22:204–206. 30581927 10.1016/j.dib.2018.12.009 PMC6301972

[41] Wang X, Zhang HG, Xiong X, Hong C, Weber GM, Brat GA, Bonzel CL, Luo Y, Duan R, Palmer NP, et al. SurvMaximin: Robust federated approach to transporting survival risk prediction models. J Biomed Inform. 2022;134:104176. 36007785 10.1016/j.jbi.2022.104176 PMC9707637

[42] Zhu F, Zhong R, Li F, Li C, Din N, Sweidan H, Potluri LB, Xiong S, Li J, Cheng B, et al. Development and validation of a deep transfer learning-based multivariable survival model to predict overall survival in lung cancer. Transl Lung Cancer Res. 2023;12(3):471–482. 37057112 10.21037/tlcr-23-84 PMC10088002

[43] OpenReview. https://openreview.net/about [accessed 8 March 2025].

[44] Kessler I, Lifshitz O, Benaim S, Wolf L*.* Cross-domain relation adaptation. Paper presented at: Proceedings of the 15th Asian Conference on Machine Learning; 2024 Dec 5–8; Hanoi, Vietnam.

[45] Holder AL, Shashikumar SP, Wardi G, Buchman TG, Nemati S. A locally optimized data-driven tool to predict sepsis-associated vasopressor use in the ICU. Crit Care Med. 2021;49(12):e1196–e1205. 34259450 10.1097/CCM.0000000000005175 PMC8602707

[46] Wu Q, Wang C, Chen Y. Heterogeneous latent transfer learning in Gaussian graphical models. Biometrics. 2024;80(3):ujae096. 39302138 10.1093/biomtc/ujae096 PMC11413907

[47] Li M, Li X, Pan K, Geva A, Yang D, Sweet SM, Bonzel CL, Ayakulangara Panickan V, Xiong X, Mandl K, et al. Multisource representation learning for pediatric knowledge extraction from electronic health records. NPJ Digit Med. 2024;7(1):319. 39533050 10.1038/s41746-024-01320-4 PMC11558010

[48] Li S, Zhang L, Cai TT, Li H. Estimation and inference for high-dimensional generalized linear models with knowledge transfer. J Am Stat Assoc. 2023;119(546):1274–1285. 38948492 10.1080/01621459.2023.2184373 PMC11213555

[49] Liu K, Zhang X, Chen W, Yu ASL, Kellum JA, Matheny ME, Simpson SQ, Hu Y, Liu M. Development and validation of a personalized model with transfer learning for acute kidney injury risk estimation using electronic health records. JAMA Netw Open. 2022;5(7): Article e2219776. 35796212 10.1001/jamanetworkopen.2022.19776 PMC9250052

[50] Tian Y, Chen W, Zhou T, Li J, Ding K, Li J. Establishment and evaluation of a multicenter collaborative prediction model construction framework supporting model generalization and continuous improvement: A pilot study. Int J Med Inform. 2020;141:104173. 32531725 10.1016/j.ijmedinf.2020.104173

[51] Steingrimsson JA, Gatsonis C, Li B, Dahabreh IJ. Transporting a prediction model for use in a new target population. Am J Epidemiol. 2023;192(2):296–304. 35872598 10.1093/aje/kwac128 PMC11004796

[52] Tian P, Chan TH, Wang Y-F, Yang W, Yin G, Zhang YD. Multiethnic polygenic risk prediction in diverse populations through transfer learning. Front Genet. 2022;13:906965. 36061179 10.3389/fgene.2022.906965 PMC9438789

[53] Wu Q, Pajor NM, Lu Y, Wolock CJ, Tong J, Lorman V, Johnson KB, Moore JH, Forrest CB, Asch DA, et al. A latent transfer learning method for estimating hospital-specific post-acute healthcare demands following SARS-CoV-2 infection. Patterns. 2024;5(11):101079. 39568467 10.1016/j.patter.2024.101079 PMC11573960

[54] Li Y, Vinzamuri B, Reddy CK. Constrained elastic net based knowledge transfer for healthcare information exchange. Data Min Knowl Disc. 2015;29(4):1094–1112.

[55] Mourragui SM, Loog M, Vis DJ, Moore K, Manjon AG, van de Wiel MA, Reinders MJT, Wessels LFA. Predicting patient response with models trained on cell lines and patient-derived xenografts by nonlinear transfer learning. Proc Natl Acad Sci USA. 2021;118: Article e2106682118. 34873056 10.1073/pnas.2106682118 PMC8670522

[56] Karbalayghareh A, Qian X, Dougherty ER. Optimal Bayesian transfer learning for count data. IEEE/ACM Trans Comput Biol Bioinform. 2019;18:644–655. 10.1109/TCBB.2019.2920981 31180899

[57] Lee T, Wollstein G, Madu CT, Wronka A, Zheng L, Zambrano R, Schuman JS, Hu J. Reducing ophthalmic health disparities through transfer learning: A novel application to overcome data inequality. Transl Vis Sci Technol. 2023;12:2. 10.1167/tvst.12.12.2 PMC10697175 38038606

[58] Wang L, Wang X, Liao KP, Cai T. Semisupervised transfer learning for evaluation of model classification performance. Biometrics. 2024;80(1):ujae002. 38465982 10.1093/biomtc/ujae002 PMC10926267

[59] Li BS, Cai T, Duan R. Targeting underrepresented populations in precision medicine: A federated transfer learning approach. Ann Appl Stat. 2023;17(4):2970–2992. 39314265 10.1214/23-AOAS1747 PMC11417462

[60] Zou N, Huang X. Empirical Bayes transfer learning for uncertainty characterization in predicting Parkinson’s disease severity. IISE Trans Healthc Syst Eng. 2018;8(3):209–219.

[61] Mayampurath A, Bashiri F, Hagopian R, Venable L, Carey K, Edelson D, Churpek M. American Heart Association’s Get With The Guidelines®-Resuscitation Investigators. Predicting neurological outcomes after in-hospital cardiac arrests for patients with coronavirus disease 2019. Resuscitation. 2022;178:55–62. 35868590 10.1016/j.resuscitation.2022.07.018 PMC9295318

[62] Ying JJ-C, Chang Y-T, Chen H-H, Chao WC. Model establishment of cross-disease course prediction using transfer learning. Appl Sci. 2022;12(10):4907.

[63] Pappy G, Aczon M, Wetzel R, Ledbetter D. Predicting high flow nasal cannula failure in an intensive care unit using a recurrent neural network with transfer learning and input data perseveration: Retrospective analysis. JMIR Med Inform. 2022;10(3): Article e31760. 35238792 10.2196/31760 PMC8931642

[64] Kim S, Kim K, Choe J, Lee I, Kang J. Improved survival analysis by learning shared genomic information from pan-cancer data. Bioinformatics. 2020;36(Supplement_1):i389–i398. 32657401 10.1093/bioinformatics/btaa462 PMC7355236

[65] Zhai J, Liu H. Cross-domain feature disentanglement for interpretable modeling of tumor microenvironment impact on drug response. IEEE J Biomed Health Inform. 2024;28(7):4382–4392. 38607708 10.1109/JBHI.2024.3387930

[66] Alatrany AS, Khan W, Hussain AJ, Mustafina J, al-Jumeily D. Transfer learning for classification of Alzheimer’s disease based on genome wide data. IEEE/ACM Trans Comput Biol Bioinform. 2023;20(5):2700–2711. 37018274 10.1109/TCBB.2022.3233869

[67] Liao H, Shang J, Sun Y. GDmicro: Classifying host disease status with GCN and deep adaptation network based on the human gut microbiome data. Bioinformatics. 2023;39(12): Article btad747. 38085234 10.1093/bioinformatics/btad747 PMC10749762

[68] Zeng X, Linwood SL, Liu C. Pretrained transformer framework on pediatric claims data for population specific tasks. Sci Rep. 2022;12(1):3651. 35256645 10.1038/s41598-022-07545-1 PMC8901645

[69] Pellegrini C, Navab N, Kazi A. Unsupervised pre-training of graph transformers on patient population graphs. Med Image Anal. 2023;89:102895. 37473609 10.1016/j.media.2023.102895

[70] Hur K, Oh J, Kim J, Kim J, Lee MJ, Cho E, Moon SE, Kim YH, Atallah L, Choi E. GenHPF: General healthcare predictive framework for multi-task multi-source learning. IEEE J Biomed Health Inform. 2024;28(1):502–513. 10.1109/JBHI.2023.3327951 37889829

[71] Khatua D, Sekh AA, Kutum R, Mukherji M, Prasher B, Kar S. Classification of Ayurveda constitution types: A deep learning approach. Soft Comput. 2023;27:5309–5317.

[72] Tang Y-C, Powell RT, Gottlieb A. Molecular pathways enhance drug response prediction using transfer learning from cell lines to tumors and patient-derived xenografts. Sci Rep. 2022;12(1):16109. 36168036 10.1038/s41598-022-20646-1 PMC9515168

[73] Sharifi-Noghabi H, Harjandi PA, Zolotareva O, Collins CC, Ester M. Out-of-distribution generalization from labelled and unlabelled gene expression data for drug response prediction. Nat Mach Intell. 2021;3:962–972.

[74] Zhang X, Xue Y, Su X, Chen S, Liu K, Chen W, Liu M, Hu Y. A transfer learning approach to correct the temporal performance drift of clinical prediction models: Retrospective cohort study. JMIR Med Inform. 2022;10(11): Article e38053. 36350705 10.2196/38053 PMC9685506

[75] Xu W, Wang T, Wang N, Zhang H, Zha Y, Ji L, Chu Y, Ning K. Artificial intelligence-enabled microbiome-based diagnosis models for a broad spectrum of cancer types. Brief Bioinform. 2023;24(3):bbad178. 37141141 10.1093/bib/bbad178

[76] Haas K, Miled ZB, Mahoui M. Medication adherence prediction through online social forums: A case study of fibromyalgia. JMIR Med Inform. 2019;7(2): Article e12561. 30946020 10.2196/12561 PMC6470459

[77] Li S, Cai TT, Li H. Transfer learning in large-scale gaussian graphical models with false discovery rate control. J Am Stat Assoc. 2023;118:2171–2183. 38143788 10.1080/01621459.2022.2044333 PMC10746133

[78] Alaa AM, Yoon J, Hu S, van der Schaar M. Personalized risk scoring for critical care prognosis using mixtures of Gaussian processes. IEEE Trans Biomed Eng. 2018;65(1):207–218. 28463183 10.1109/TBME.2017.2698602

[79] Turki T, Wei Z, Wang JTL. A transfer learning approach via procrustes analysis and mean shift for cancer drug sensitivity prediction. J Bioinforma Comput Biol. 2018;16(3):1840014. 10.1142/S0219720018400140 29945499

[80] Dhruba SR, Rahman R, Matlock K, Ghosh S, Pal R. Application of transfer learning for cancer drug sensitivity prediction. BMC Bioinformatics. 2018;19(Suppl 17):497. 30591023 10.1186/s12859-018-2465-y PMC6309077

[81] Estiri H, Vasey S, Murphy SN. Generative transfer learning for measuring plausibility of EHR diagnosis records. J Am Med Inform Assoc. 2021;28:559–568. 33043366 10.1093/jamia/ocaa215 PMC7936395

[82] Ismailoglu F, Cavill R, Smirnov E, Zhou S, Collins P, Peeters R. Heterogeneous domain adaptation for IHC classification of breast cancer subtypes. IEEE/ACM Trans Comput Biol Bioinform. 2018;17(1):347–353. 30369448 10.1109/TCBB.2018.2877755

[83] Mignone P, Pio G, Ceci M. Distributed heterogeneous transfer learning. Big Data Research. 2024;37:100456.

[84] Chiu H-J, Li T-HS, Kuo P-H. Breast cancer–detection system using PCA, multilayer perceptron, transfer learning, and support vector machine. IEEE Access. 2020;8(3):204309–204324.

[85] Li J, Tian Y, Li R, Zhou T, Li J, Ding K, Li J. Improving prediction for medical institution with limited patient data: Leveraging hospital-specific data based on multicenter collaborative research network. Artif Intell Med. 2021;113:102024. 33685587 10.1016/j.artmed.2021.102024

[86] Meng X, Wang X, Zhang X, Zhang C, Zhang Z, Zhang K, Wang S. A novel attention-mechanism based Cox survival model by exploiting pan-cancer empirical genomic information. Cells. 2022;11(9):1421. 35563727 10.3390/cells11091421 PMC9100007

[87] Przedborski M, Smalley M, Thiyagarajan S, Goldman A, Kohandel M. Systems biology informed neural networks (SBINN) predict response and novel combinations for PD-1 checkpoint blockade. Commun Biol. 2021;4(1):877. 34267327 10.1038/s42003-021-02393-7 PMC8282606

[88] Hai AA, Weiner MG, Livshits A, Brown JR, Paranjape A, Hwang W, Kirchner LH, Mathioudakis N, French EK, Obradovic Z, et al. Domain generalization for enhanced predictions of hospital readmission on unseen domains among patients with diabetes. Artif Intell Med. 2024;158:103010. 39556977 10.1016/j.artmed.2024.103010 PMC11602339

[89] Lim H, Kim G, Choi J-H. Advancing diabetes prediction with a progressive self-transfer learning framework for discrete time series data. Sci Rep. 2023;13(1):21044. 38030750 10.1038/s41598-023-48463-0 PMC10687240

[90] Toseef M, Li X, Wong K-C. Reducing healthcare disparities using multiple multiethnic data distributions with fine-tuning of transfer learning. Brief Bioinform. 2022;23(3):bbac078. 35323862 10.1093/bib/bbac078

[91] Zhao Z, Fritsche LG, Smith JA, Mukherjee B, Lee S. The construction of cross-population polygenic risk scores using transfer learning. Am J Hum Genet. 2022;109(11):1998–2008. 36240765 10.1016/j.ajhg.2022.09.010 PMC9674947

[92] Zhang H, Chong H, Yu Q, Zha Y, Cheng M, Ning K. Tracing human life trajectory using gut microbial communities by context-aware deep learning. Brief Bioinform. 2023;24(1):bbac629. 36631408 10.1093/bib/bbac629

[93] Chen J, Chen Y, Li J, Wang J, Lin Z, Nandi AK. Stroke risk prediction with hybrid deep transfer learning framework. IEEE J Biomed Health Inform. 2021;26(1):411–422. 10.1109/JBHI.2021.3088750 34115602

[94] Lemmon J, Guo LL, Steinberg E, Morse KE, Fleming SL, Aftandilian C, Pfohl SR, Posada JD, Shah N, Fries J, et al. Self-supervised machine learning using adult inpatient data produces effective models for pediatric clinical prediction tasks. J Am Med Inform Assoc. 2023;30(12):2004–2011. 37639620 10.1093/jamia/ocad175 PMC10654865

[95] Gao Y, Cui Y. Optimizing clinico-genomic disease prediction across ancestries: A machine learning strategy with Pareto improvement. Genome Med. 2024;16(1):76. 38835075 10.1186/s13073-024-01345-0 PMC11149372

[96] Kumar Y, Ilin A, Salo H, Kulathinal S, Leinonen MK, Marttinen P. Self-supervised forecasting in electronic health records with attention-free models. IEEE Trans Artif Intell. 2024;5(8):3926–3938.

[97] Chai H, Zhang Z, Wang Y, Yang Y. Predicting bladder cancer prognosis by integrating multi-omics data through a transfer learning-based cox proportional hazards network. CCF Trans High Perform Comput. 2021;3:311–319.

[98] Cai T, Li M, Liu M. Semi-supervised triply robust inductive transfer learning. J Am Stat Assoc. 2025;120(550):1037–1047. 40519952 10.1080/01621459.2024.2393463 PMC12165252

[99] Lu Y, Gu T, Duan R. Enhancing genetic risk prediction through federated semi-supervised transfer learning with inaccurate electronic health record data. Stat Biosci. 2024. 10.1007/s12561-024-09449-2 PMC12409711 40917581

[100] Kuru HI, Cicek AE, Tastan O. From cell lines to cancer patients: Personalized drug synergy prediction. Bioinformatics. 2024;40(5):btae134. 10.1093/bioinformatics/btae134 PMC11215552 38718189

[101] Zheng X, Meng D, Chen D, Wong WK, To KH, Zhu L, Wu J, Liang Y, Leung KS, Wong MH, et al. scCaT: An explainable capsulating architecture for sepsis diagnosis transferring from single-cell RNA sequencing. PLOS Comput Biol. 2024;20(10): Article e1012083. 39432561 10.1371/journal.pcbi.1012083 PMC11527285

